# Dps Functions as a Key Player in Bacterial Iron Homeostasis

**DOI:** 10.1021/acsomega.3c03277

**Published:** 2023-09-11

**Authors:** Sunanda Margrett Williams, Dipankar Chatterji

**Affiliations:** † Institute of Structural and Molecular Biology, Birkbeck, University of London, Malet Street, London WC1E 7HX, United Kingdom; ‡ Molecular Biophysics Unit, Indian Institute of Science, Bangalore 560012, India

## Abstract

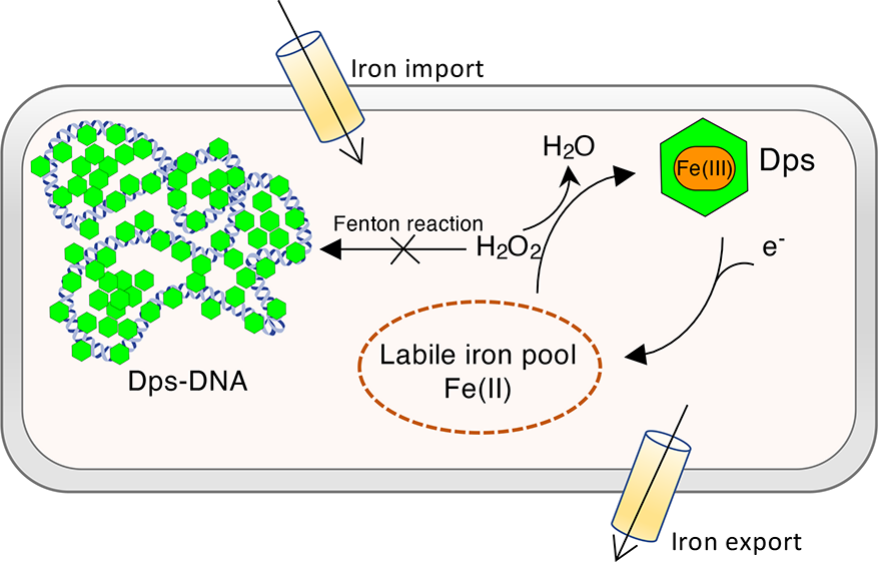

Iron plays a vital role in the maintenance of life, being central to various cellular processes, from respiration to gene regulation. It is essential for iron to be stored in a nontoxic and readily available form. DNA binding proteins under starvation (Dps) belong to the ferritin family of iron storage proteins and are adept at storing iron in their hollow protein shells. Existing solely in prokaryotes, these proteins have the additional functions of DNA binding and protection from oxidative stress. Iron storage proteins play a functional role in storage, release, and transfer of iron and therefore are central to the optimal functioning of iron homeostasis. Here we review the multifarious properties of Dps through relevant biochemical and structural studies with a focus on iron storage and ferroxidation. We also examine the role of Dps as a possible candidate as an iron donor to iron–sulfur (Fe–S) clusters, which are ubiquitous to many biological processes.

## 1 Introduction

Iron plays a central role in the biology of living organisms and is the fourth most abundant element in the Earth’s crust. It is also a versatile element which can act as both an electron donor and acceptor, and in biological systems this modulates the transition between the forms Fe^2+^ (ferrous), Fe^3+^ (ferric), and Fe^4+^ (ferryl). This redox chemistry endows iron with an important role in vital biological processes due to its ability to act as a cofactor in many redox processes and therefore is incorporated in the active site of many enzymes.^1,2^ But free iron poses a problem for aerobic organisms, as it is easily oxidized to Fe^3+^, which is insoluble and nonbioavailable. This process also generates toxic hydroxyl radicals that are harmful to living organisms.^3^

The functional role of iron is often dependent on its association with proteins. Iron is the most common redox active metal within prosthetic groups found in proteins, such as heme or iron–sulfur clusters. Iron binding proteins are crucial for fundamental cellular processes such as respiration, metabolism, and DNA repair. Free iron has the potential to catalyze damage to DNA, proteins, and lipids though its participation in Fenton chemistry. Free ferrous iron also can react with H_2_O_2_ to produce reactive species such as hydroxyl radicals and ferric iron.^4^ The iron-induced oxidative stress could be further exacerbated by the iron released from iron binding proteins during attack by reactive species. To circumvent the apparent paradox of iron limitation and iron toxicity, the concentration of chelatable iron which ensures correct metalation of iron containing proteome while minimizing the possibility of iron-induced ROS generation, is maintained through a process termed iron homeostasis.^5,6^

In this review we look at the role of iron in oxidative stress in prokaryotes and the ensemble of defense mechanisms available to living cells. A crucial role in iron homeostasis is played by Dps (DNA binding proteins in starved cells) in prokaryotes. We also look at the emerging evidence of interplay between iron storage proteins such as Dps to regulate iron access to Fe–S cluster proteins and protect them from oxidative stress.

## 2 Oxidative Stress and Iron Homeostasis

Oxygen is used by most living organisms, with the exception of anaerobic bacteria, to generate energy in the form of ATP by oxidative phosphorylation.^3^ Iron is a dangerous metal in this oxygenated environment due to its capacity to generate reactive oxygen species (ROS) such as superoxide (O_2_^•–^), hydrogen peroxide (H_2_O_2_), and the highly destructive hydroxyl radical (^•^OH). It is important for bacteria to have tight control over iron uptake and store iron to restrict ROS buildup but at the same time maintain optimum iron levels in cells for survival.

Thus, there is an intimate relationship between iron homeostasis and response to oxidative stress.^6^ To achieve this, bacteria use iron-dependent global regulators to sense iron availability in the cell and regulate the expression of proteins involved in iron acquisition, storage, and efflux, accordingly.^7^ This section details how iron contributes to oxidative stress and illustrates approaches evolved by bacteria to overcome the twin problems of insolubility and the toxic potential of free iron.

### Iron-Mediated Oxidative Stress

Oxidative stress is a phenomenon caused by an imbalance in production and accumulation of ROS and the ability to detoxify these reactive species. Molecular oxygen (O_2_) is highly reactive, with two spin-aligned unpaired electrons in its π antibonding orbitals. Consequently, the unpaired electrons of dioxygen react with the unpaired electron of transition metals, like iron (Reaction 1):^8^
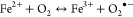
The superoxide anion (O_2_^•–^), a byproduct of respiration and photosynthesis, could also drive the backward reaction with Fe^3+^ (ferric iron) to generate Fe^2+^ (ferrous iron).

H_2_O_2_, another byproduct of oxidative respiration, reacts with Fe^2+^ (ferrous iron) to generate hydroxyl free radicals through a process termed Fenton reaction (Reaction 2):^9^
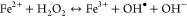
Through Reactions 1 and 2, iron catalyzes the Haber–Weiss reaction, resulting in the production of hydroxyl radicals (Reaction 3):
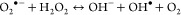
The Fenton chemistry is linked to protein carbonylation and membrane peroxidation and has a negative impact on DNA. A 10 min exposure to millimolar levels of H_2_O_2_ has been reported to cause enough DNA damage to heavily mutate or kill most bacteria.^10^

In living cells iron is predominantly associated with proteins but also exists as a pool of “labile iron” which indicates the redox potential of iron and its availability to exchange between ligands and chelators.^11^ Iron, which is the only metal implicated in Fenton reaction to date, is this “free iron” in the labile pool which is not incorporated into enzymes or iron-storage proteins. Cu(I) can transfer electrons to H_2_O_2_, but its impact *in vivo* is negligible as the levels of copper in its labile pool are finely controlled due to its high toxicity.^12,13^

Cells have iron chelators to prevent this iron-mediated damage to biomolecules. It is therefore not surprising that bacteria respond to instances of H_2_O_2_ accumulation by the induction of a ferritin-like protein called Dps, a scavenger of free iron.^14^

### Iron Homeostasis: Bacterial Management of Iron

In this section, we describe the elaborate mechanisms of iron regulation that enable cells to acquire enough iron for survival and maintain low levels of “free” iron which could potentially cause stress and damage by pathways described earlier. There is a requirement to adapt defenses against oxidative stress to the iron in environment and “sense” iron as a signal for potential oxidative stress.^15^

#### Iron Acquisition by Cells

An *E. coli* cell has been shown to contain up to 10^5^–10^6^ iron atoms per cell depending on its stage of growth.^16^ Due to the low solubility of ferric iron (Fe^3+^), it is generally acquired in its reduced ferrous (Fe^2+^) form, or the ferric form is made more soluble by lowering the external pH. Another widely used strategy is to employ ferric ion chelators like siderophores as solubilizing agents that acquire iron from the external environment.^17^ These are low molecular mass compounds (150–2000 Da) with specificity for ferric iron with over 500 characterized examples. They fall into three classes, the catechols, hydroxamates, and α-hydroxycarboxylates based on the nature of the iron-ligating moiety.^18^ Once siderophores are secreted from cells, they acquire iron by competing with host proteins (in the case of pathogens) or by solubilization of Fe^3+^ from iron containing minerals.

The Fe(III)–siderophore complexes require outer membrane (OM) receptor proteins that bind with high specificity, followed by active translocation through the plasma membrane by an ABC transporter.^19^ These OM siderophore receptors are induced by iron starvation with multiple OM receptors specific for different siderophores found in bacteria. Once inside, the Fe(III)–siderophore complexes are reduced, leading to dissociation of Fe(II) which has relatively low affinity for siderophores.^20^ But in the case of hexadentate triscatechelates, which form high affinity iron complexes, iron release pathways comprise the hydrolysis of the siderophore backbone and further reduction of Fe(III) to Fe(II) by intracellular reductants.^7,21^

Under acidic or anaerobic conditions, iron uptake is predominantly in the soluble ferrous (Fe^2+^) state, and bacteria have evolved mechanisms for direct uptake of ferrous iron. In Gram-negative bacteria, the soluble ferrous iron enters the periplasm by free diffusion through porins where it is transported into cytoplasm via different transport systems such as MntH, ZupT, YfeABCD, FutABC, EfeUOB, and Feo. Of these, EfeUOB^22^ and FeoABC transporters are the only bacterial systems solely dedicated to transport of ferrous iron. The transport system EfeUOB has been found only in pathogenic species, whereas Feo^23^ is the main ferrous iron transport system that is present in pathogenic species as well as in nonpathogenic microbes.^24^

For bacterial pathogens, iron restriction is even more extreme as the iron availability in the host is limited by sequestration of iron within intracellular proteins like hemoglobin, cytochromes, or dedicated iron storage proteins such as ferritins and chelating extracellular Fe^3+^ with glycoproteins such as transferrins and lactoferrins.^25^ There are two main ways through which pathogens acquire iron from the host: by direct contact of the bacterium with host iron sources such as transferrins and heme proteins or by employing siderophores which capture iron from host transferrin and ferritin proteins.

#### Iron Storage Proteins

**Figure 1:**
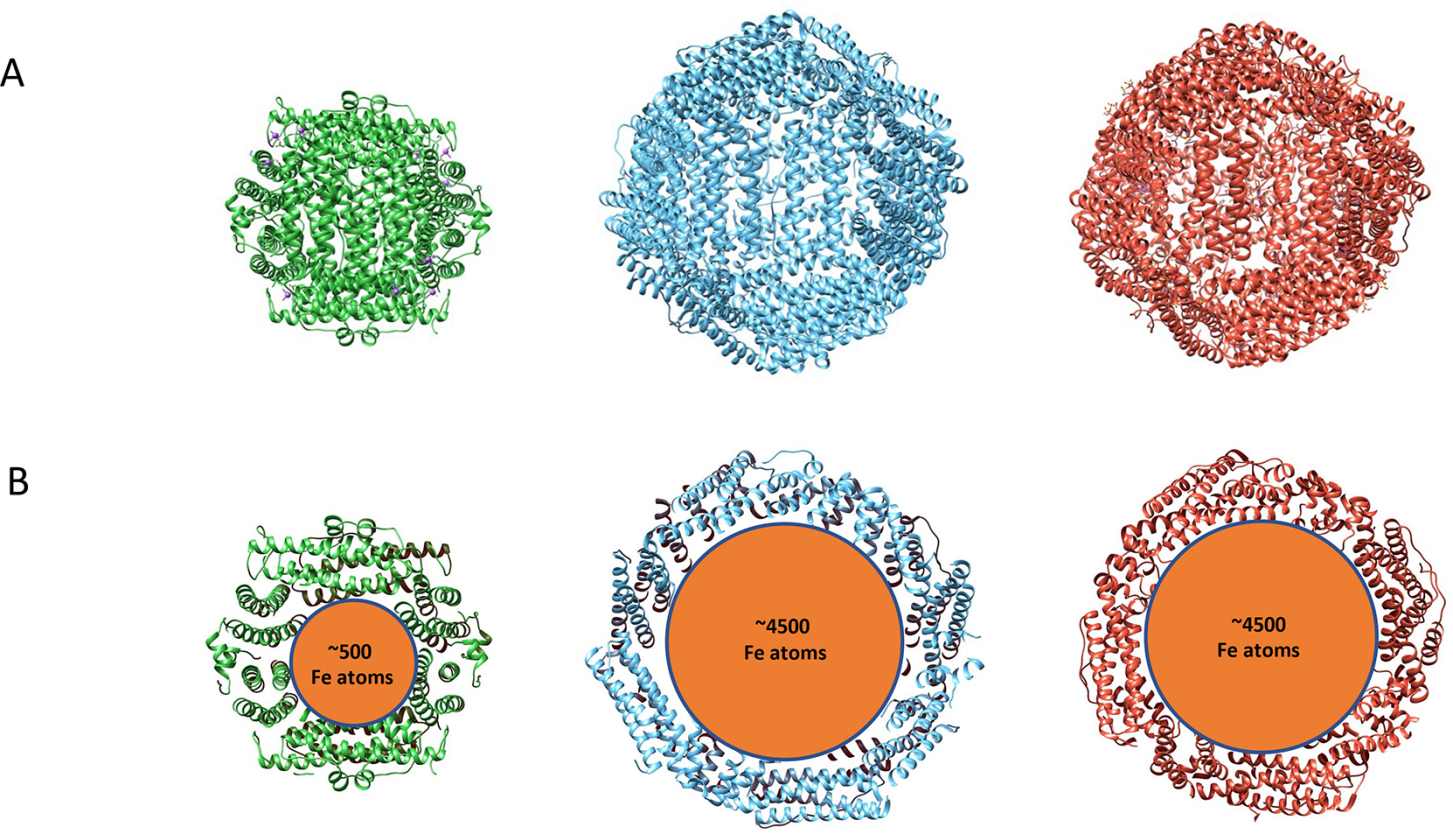
X-ray structures of *E. coli* (A) Dps in green (PDB ID: 1dps), ferritin in light blue (PDB ID: 1eum), and bacterioferritin in red (PDB ID: 3e1j). (B) Cross-sectional views showing the central cavity available for iron storage. Ferritins and bacterioferritins have a capacity of around 4500 iron atoms per molecule, whereas the smaller Dps shell has a capacity of around 500 iron atoms.

Apart from acquiring iron extracellularly, bacteria store intracellular iron reserves within dedicated iron storage proteins belonging to the ferritin super family, found in all kingdoms of life.^26^ These proteins store iron in a nonreactive state, which can be remobilized to satisfy cellular requirements during conditions of iron starvation. In bacteria there are three types of cage-forming iron storage proteins, namely, the universal ferritins, the heme containing bacterioferritins seen in eubacteria, and the smaller Dps present only in prokaryotes. They are composed of identical or similar alpha helical bundles, either 24 (ferritins and bacterioferritins) or 12 (Dps) in number, that assemble to form a roughly spherical protein shell surrounding a central cavity which acts as an iron storage reservoir (Figure 1).^27^

The larger ferritins and bacterioferritins can accommodate around 4500 iron atoms per 24-mer, whereas the smaller Dps have a lower storage capacity of around 500 iron atoms per 12-mer.^28^ These proteins contain catalytic centers where the soluble Fe(II) form is oxidized to Fe(III) and deposited in the cavity as a ferric mineral.^29^ The pathway of iron release from ferrin-like proteins is less well-known, but more evidence has emerged in recent years. In eukaryotic ferritins, electrons shuttled from NADPH through a flavin-nucleotide carries out the reduction of Fe^3+^ to Fe^2+^, triggering release of iron from the mineral store.^30^ In bacterioferritins from *P. aeruginosa*, a bacterioferritin-associated ferredoxin (Bfd) promotes mobilization of stored iron by binding to BfrB.^31^ Ferredoxins are also thought to play a role in iron release of *N. punctiforme* Dps.^32^

**Figure 2:**
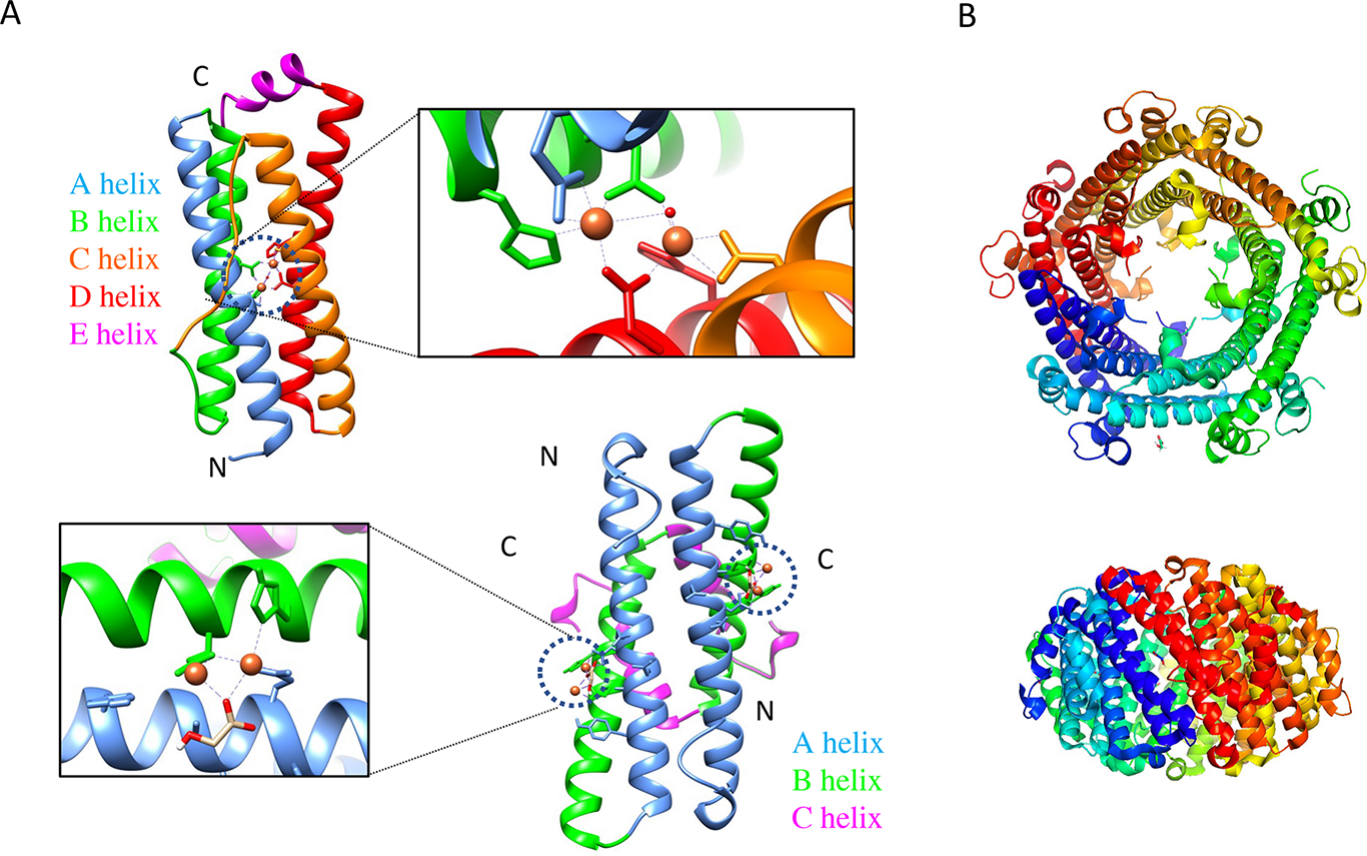
(A) Top panel: X-ray structure of *E. coli* ferritin monomer (PDB ID: 1eum) showing a typical ferritin-fold characterized by a four helical bundle, made up of two homologous pairs of antiparallel alpha helices arranged in an up–down–down–up topology. The intrasubunit ferroxidation site is indicated with a dashed black circle, and the expanded image is shown to the right. Fe ions are in orange. The color coding of the helices from A–E is as in the labels. Bottom panel: X-ray structure of *Rhodospirillum rubrum* encapsulated ferritin (PDB ID: 5DA5) showing a dimer formed of two monomeric subunits of antiparallel alpha helices, color coded A–C as indicated in the label. The ferroxidation sites (one per monomer) are in dashed circles, zoomed in on the rectangle on the left. (B) Top view of the EncFtn pentamer of dimer (decamer) arrangement in the top panel and side view in the bottom panel. The decamer is 7 nm in diameter with a thickness of 4.5 nm (PDB ID: 5DA5).

Another mode of storage is using encapsulins, which are large macromolecular assemblies similar to viral capsids, widespread in bacteria and archaea. Encapsulins as their name suggests can encapsulate proteins targeted to the capsid via short C-terminal signal sequences present on the cargo proteins.^33^ A class of ferritin superfamily proteins called encapsulated ferritins (EncFtn) is among the cargo proteins of encapsulins. EncFtn assembles to form annular pentamers of dimers different in architecture from their cage-forming counterparts. They have ferroxidase activity but lack the intrinsic ability to solubilize mineral cores unless localized within encapsulins. They can store around four times the amount of iron compared to classical ferritins due to their association with the larger encapsulins.^34^ The differences in EncFtn relative to canonical ferritins are illustrated in Figure 2.

#### Redox Stress Defense Systems

Defense mechanisms against redox stress aim to keep the concentration of ROS at nonlethal levels or repair damage due to instances of oxidative damage. In bacteria, scavenger enzymes that consume ROS like superoxide dismutases (SODs), catalases, and peroxidases are critical in self-defense mechanisms against oxidative stress. *E. coli* has two cytoplasmic SODs, one containing iron (Fe-SOD) and the other manganese (Mn-SOD) which convert superoxide (O_2_^•–^) to H_2_O_2_ and O_2_.^15^ In Gram-negative bacteria, a periplasmic SOD (Cu–Zn–SOD) protects from superoxides outside the cytoplasmic membrane which may escape from the respiratory chain components.^35^

H_2_O_2_ is removed by catalases and peroxidases, yielding H_2_O and O_2_. Catalases act at higher levels of H_2_O_2_ or under starvation conditions, whereas peroxidases are the primary scavengers at lower H_2_O_2_ concentrations.^8^ Nonenzymatic antioxidants like NADPH and NADH pools, β-carotene, ascorbic acid, α-tocopherol, and glutathione maintained in their reduced state by glutathione reductase, are constitutively present and help to maintain an intracellular reducing environment or scavenge ROS.^36^

Genetic responses that greatly increase the resistance of the cells also occur in bacteria triggered by oxidative stress. These are through transcriptional regulators which act to regulate antioxidant systems in response to perceiving redox signals from ROS. The two-component SoxRS regulon responds to O_2_^•–^ and to redox-cycling compounds but not to H_2_O_2_.^37^ The SoxRS regulon activates at least 15 genes including Mn-SOD, endonuclease IV, ferredoxin reductase, and fumarase.^38^ The OxyR system is primarily responsible for sensing and maintaining H_2_O_2_ levels in cells. Catalase, glutathione reductase, and Dps genes are activated by the OxyR system. PerR and RitR are redox sensors prevalent in Gram-positive bacteria such as streptococci for preventing peroxide stress and are necessary for virulence.^36,39^

#### Iron Efflux from Cells

Efflux systems responsible for iron efflux to counter oxidative stress may be required in some instances. Only a few examples of efflux systems are known, and we lack understanding of their mechanistic details. Some of these efflux pumps are the membrane-bound P-type ATPases and cation diffusion facilitator (CDF) metal ion transporters which are ubiquitous among prokaryotes and eukaryotes, transporting a wide range of cations.^7^ Another class of proteins, namely, the major facilitator superfamily proteins, functions in the transmembrane transport of cations.^40^

Membrane-bound ferritins (Mbfs) composed of a cytoplasmic N-terminus containing a ferritin-like domain and a C-terminal membrane spanning domain, are also implicated in iron efflux.^41^ These lack the cage forming ability of classical ferritins but have ferroxidase activity mediated by the N-terminal ferritin-like domain. In *Agrobacterium tumefaciens* for instance, they are considered important in mediating oxidative stress response during plant infection.^42^*A. tumefaciens* mbfA null strain had 1.5-fold higher total iron content compared to the WT, and overexpression of mbfA reduced total iron content by 2-fold in the WT. Thus, although they have ferroxidase activity like ferritins, these results point to the function of MbfA as an iron exporter rather than for iron storage.^43^

## 3 Elaborating the Role of Dps in Iron Homeostasis

Dps are ferritin-like proteins with ferroxidation and DNA binding properties that afford protection during oxidative and nutritional stress. They are particularly interesting due to their nonspecific DNA binding property which enables them to protect DNA during oxidative stress.^44^ Thus, they are also classed as a nucleoid associated protein (NAP) similar to IHF, HU, H-NS, etc. These proteins are overexpressed during starvation, but constitutively expressed homologues are also seen, such as the second Dps in *M. smegmatis* (MsDps2).^45^

Dps homologues have a multifaceted role in bacteria, while retaining some of the classical properties of DNA binding and ferroxidation.^27^ The well-studied Dps from *E. coli* additionally protect the cells from UV and gamma radiations, copper toxicity, thermal stress, and acid/base shock.^46^*S. mutans*^47^ and *S. pyogenes*(48) do not produce a catalase enzyme responsible for H_2_O_2_ elimination, but the Dps homologue present in these bacteria, namely, Dpr (Dps-like peroxide resistance), confers peroxide resistance. The cyanobacterium *N. punctiforme* harbors five Dps (NpDps 1–5) having distinct features and cell-specific expression.^49^

In *E. coli*, Dps null phenotypes are viable under controlled conditions but have been shown to have significantly increased mortality rates when exposed to stress conditions like starvation, oxidative and thermal stress, metal toxicity, etc.^50^ This effect of Dps knockout has also been demonstrated in Dps from several bacteria such as *Bacillus cereus*,^51^*Deinococcus wulumuqiensis*,^52^*Porphyromonas gingivalis*,^53^ etc. The protective function of Dps under a broad array of stresses is thought to be a combination of both its iron storage and DNA binding properties which are required for full preservation of DNA integrity and biological activities.^54^ Dps was introduced in the earlier section as an iron storage protein; this section will examine them in more detail with specific emphasis on structure which is intertwined with their function.

### Iron Oxidation and Storage

Dps subunits have a ferritin-like fold that assembles into dodecamers with 23 tetrahedral symmetry with an outer diameter of 90 Å and an internal diameter of 45 Å, leaving a central hollow core where approximately 500 iron atoms can be stored per dodecamer (Figure 1). The structural basis of ferroxidation was first described in the Dps homologue from *Listeria innocua*, where iron-bound ferroxidation sites were identified. H_2_O_2_ is the preferred reagent for iron oxidation in Dps which is ∼100-fold more efficient than iron oxidation with O_2_, contrary to ferritins where in general dioxygen is the main electron acceptor.^55^ In *E. coli* Ferritin A, EcFtnA, it was shown that there are multiple iron-oxidation pathways with O_2_ and H_2_O_2_ as oxidants.^56^ Bacterioferritin could utilize both O_2_ and H_2_O_2_ for oxidation of iron,^57^ although the EcBfr (*E. coli* bacterioferritin) ferroxidase center reacts rapidly with H_2_O_2_ and at a slower rate with O_2_.^58^ Thus, ferritins and bacterioferritins could vary the reagent for iron oxidation depending on the physiological needs of the cell.

**Figure 3:**
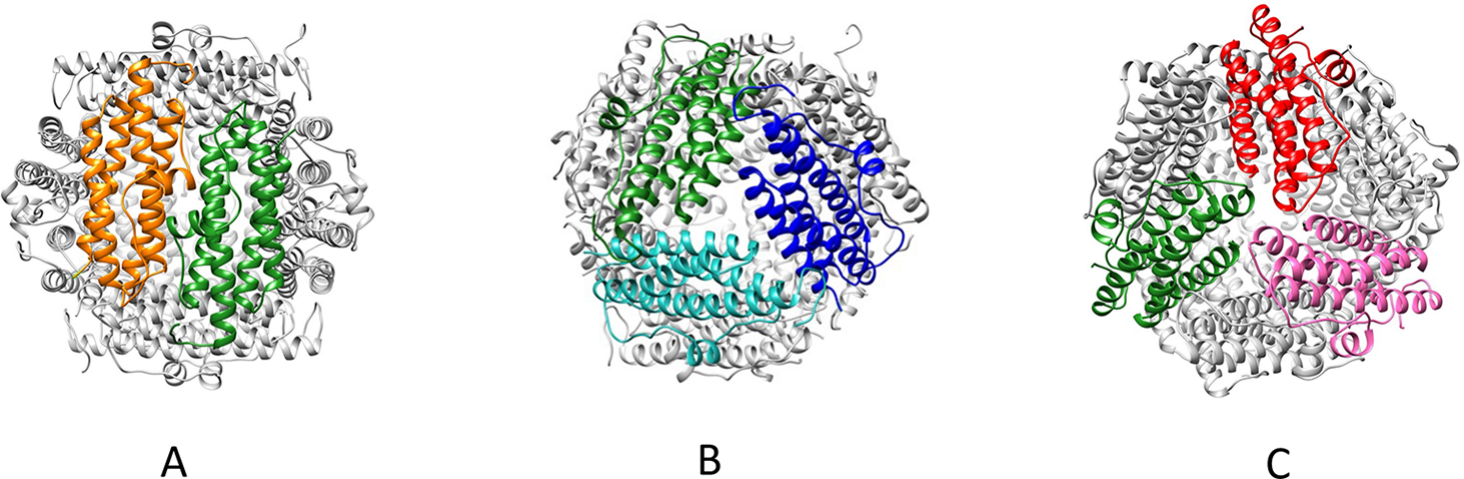
Multimerization interfaces of Dps. A Dps monomer in green color (PDB ID: 1dps) forms three types of symmetry interfaces with its neighboring homomeric subunits, namely, (A) a 2-fold interface and two types of trimeric (3-fold) interfaces, (B) ferritin-like 3-fold interface, and (C) Dps-like trimeric interface.

**Figure 4:**
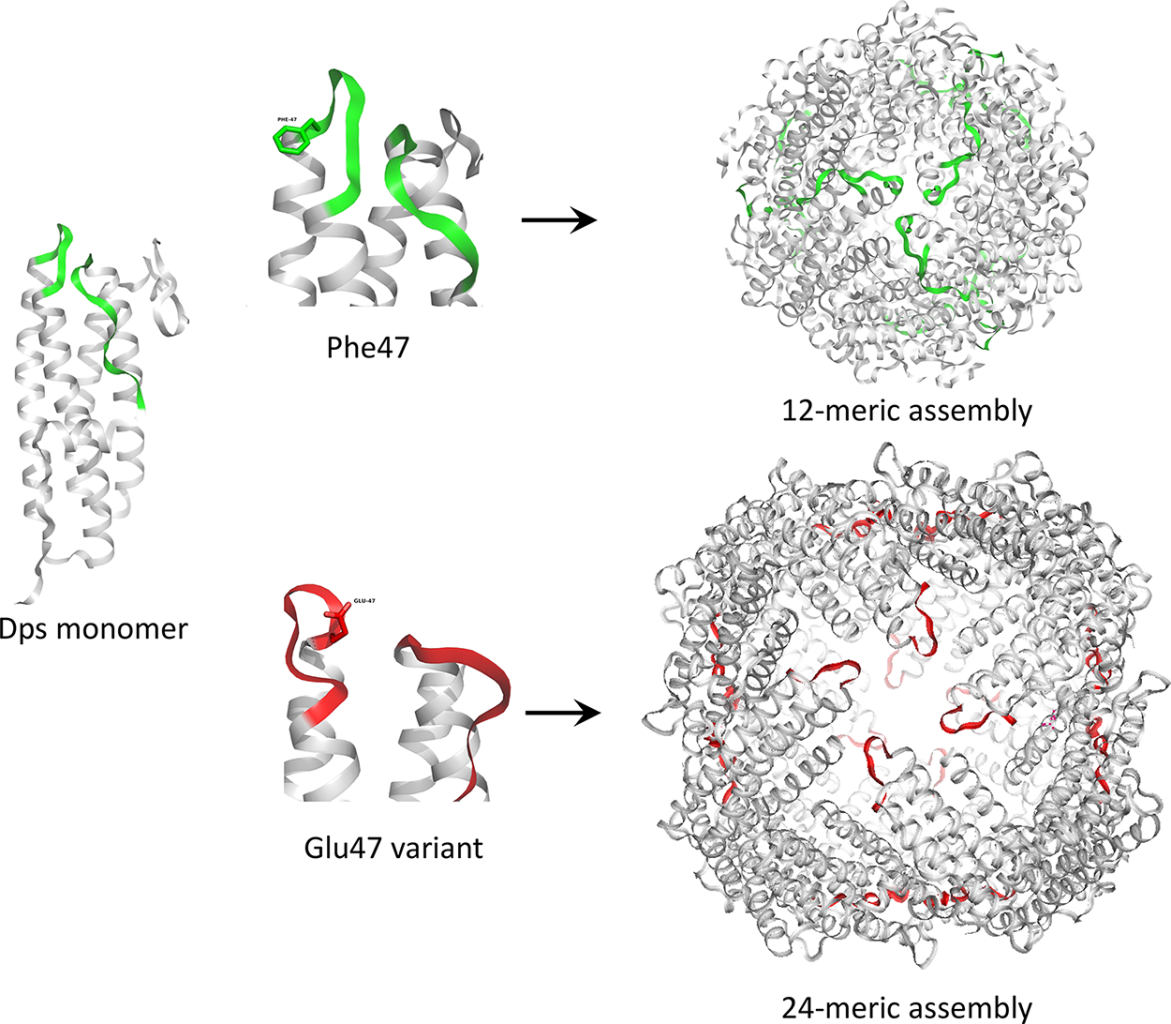
Structural switch from a 12-mer to a 24-meric assembly. A ferritin-fold is exemplified by a monomer of Dps1 from *M. smegmatis* (MsDps1) (PDB ID: 1VEI). A single point mutation in the AB loop (in green) from Phe47 (green stick representation) to Glu47 (red stick) (PDB ID: 5H46) switches the assembly to a ferritin-like 24-mer, under crystalline conditions.

Dps 12-mer has interfaces related by 2-fold and two types of 3-fold interfaces, namely, the ferritin-like and Dps-like trimeric interface (Figure 3).^59^ Ferritins are composed of 24 structurally identical subunits which assemble into a cage with 432 octahedral symmetry and therefore have a 4-fold interface in addition to the 2-fold and the ferrin-like 3-fold interfaces. Interestingly, a mutation at the Dps-like 3-fold interface on a conserved residue in a loop region switches the assembly of Dps from 12-mers to ferritin-like 24-mers under crystallization conditions (Figure 4).^59^ Thus, the alpha helical bundle which is a signature of ferritin family proteins could potentially fold into 12- or 24-meric assemblies based on the interactions that are sustained in the loop regions. The evolutionary trees of the ferritin-family proteins suggest that rubyerythrin-like ancestors evolved into 12-meric bacterioferritins which later diverged into Dps and Bfr/ferritin proteins.^60^

In Dps, iron is incorporated and oxidized in a multistep process as described below.^27,55,61^

#### Fe(II) Atoms Enter the Protein Cavity (Step 1)

The entry site for incoming Fe(II) atoms is the four channels enclosed by the ferritin-like trimeric interface lined by hydrophilic residues, such as negatively charged aspartates and glutamates. The channel is funnel-shaped with a wide mouth at the solvent side which narrows toward the interior of the protein cavity.^62^ Several studies point to these as both the entry and exit channels for iron in Dps, and substitution at these loci affected the rates of iron uptake and release.^61−63^ Several flexible aspartates are proposed to propel iron atoms from the entry sites to ferroxidase sites for oxidation.^64^

#### Fe(II) Binds to the Ferroxidase Site (Step 2)

Twenty-four Fe(II) bind at the 12 di-iron ferroxidation sites of the protein described by the equation:
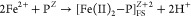
where [Fe(II)_2_–P]^*Z*+2^_FS_ denotes a di-Fe(II)–protein complex at each of the 12 ferroxidase sites.

The binding affinities of one site have a relatively high affinity for iron in the di-iron ferroxidation center, and iron binds with a lower affinity at the other site. The above equation holds true only for a full occupancy of the 12 di-iron centers which corresponds to 24Fe(II)/Dps. Under anaerobic conditions, *E. coli*, *B. subtilis*, and *L. innocua* Dps only bind 12 Fe(II)/Dps, as a bridging oxidant is required to tether the iron at high and low affinity sites. So the above equation of iron binding may not hold true in such situations.^65^

#### Fe(II) Oxidation at the Ferroxidase Site (Step 3)

Rapid pairwise oxidization of two Fe(II) by one molecule of H_2_O_2_ occurs at the dinuclear ferroxidase sites. Thus, for every two Fe(II) oxidized an H_2_O_2_ is reduced, thereby preventing the generation of hydroxyl radicals through Fenton chemistry.
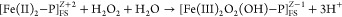


#### Nucleation and Growth of the Mineral Core (Step 4)

The Fe(III) mineral core is formed at an unknown location, with the possibility of further incoming Fe(II) atoms getting oxidized directly on the surface of this growing mineral core. A ferric core of ∼500 Fe(III) is formed inside the Dps shell, following the mineralization equation with a 2 Fe(II) per H_2_O_2_ stoichiometry:
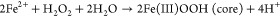
Zhao et al. proposed that the 2:1 stoichiometry of Fe(II) and H_2_O_2_ is mediated by the protein shell, or the iron core undergoes pairwise ferrous oxidation through the adsorption of H_2_O_2_ directly on the mineral surface.^55^

**Figure 5:**
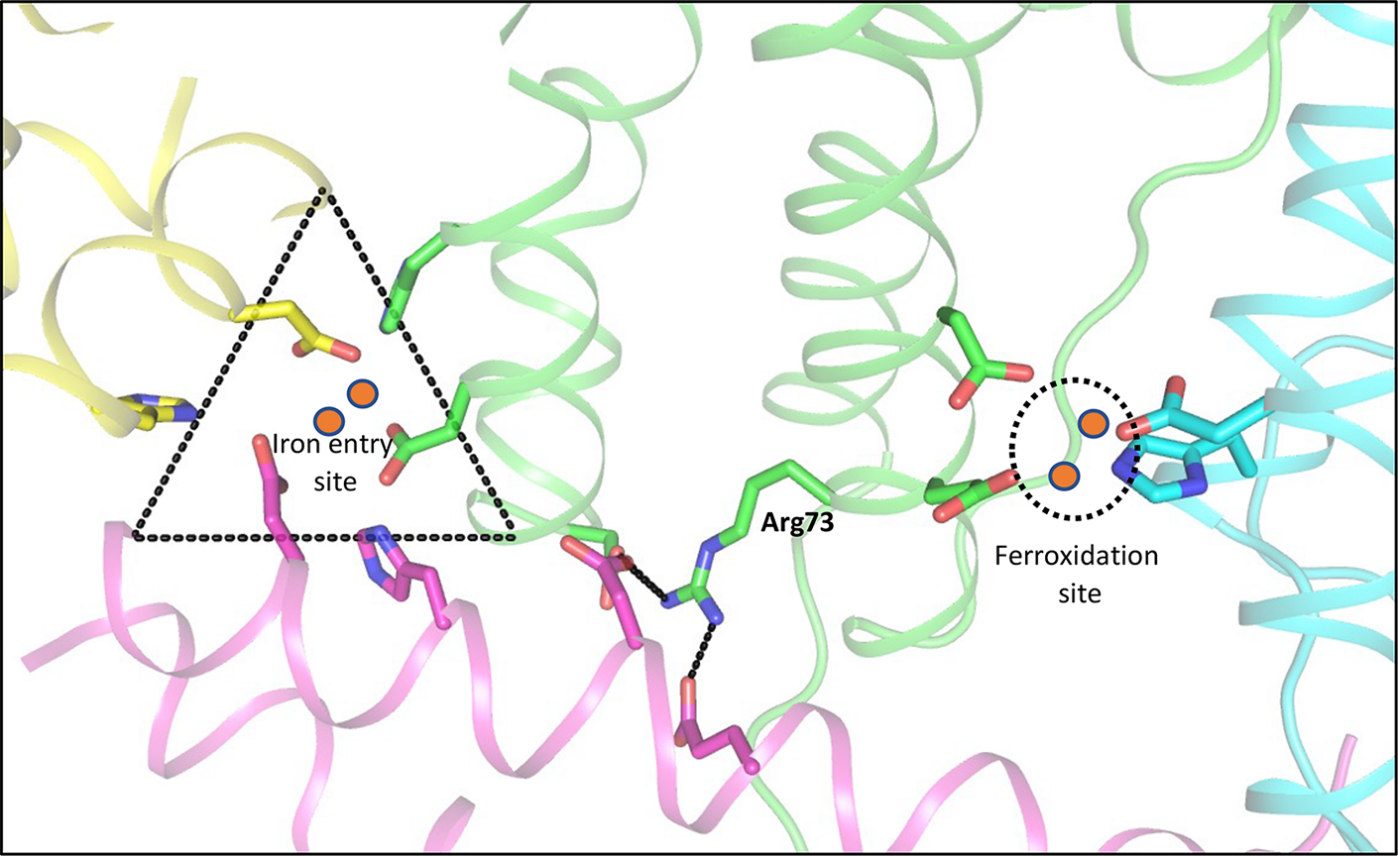
View of the internal organization of a typical Dps shell (Dps2 from *M.smegmatis*, PDB ID: 2z90) showing the iron entry sites (in dashed triangle) and the ferroxidation site (dashed circle). These lie at the interface between different subunits of the dodecamer, which are colored differently to indicate this intricate interplay. A conserved arginine (Arg73 in MsDps2) is thought to link the ferroxidation site to the iron entry site by stabilizing this interface.

The formation of the mineral core in Dps was structurally evaluated through cryoEM with iron-loaded Dps showing the presence of large Fe(III) clusters formed of 1–1.5 nm discrete subunits.^66^ The pathway for iron entry, oxidation, and storage with emphasis on the amino acid residues involved in this process is depicted in Figure 5. In Dps, ferroxidation can also take place, albeit at a much slower rate with O_2_ as an oxidant.^61^

### Nonspecific DNA Binding

DNA is the prime information molecule of the cell, and it encodes the primary structure of proteins. These macromolecules are constantly in the line of damage caused by intrinsic and extrinsic sources. The extrinsic damage to DNA includes UV radiation and environmental toxins, whereas intrinsic damage consists mainly of reactive oxygen species (ROS) and spontaneous hydrolysis.^67^ Protection mechanisms to offset these lethal effects are of considerable interest for cell survival. During conditions of nutrient depletion, energy production processes become inefficient, and the cell cannot afford energy-extravagant DNA defense mechanisms for DNA repair. For example, in starved *E. coli* cells Dps was shown to accumulate in very large amounts and formed a major component of chromosome in late stationary phase cells to carry out condensation of DNA into biocrystalline structures.^9,68^ Thus, Dps bind and compact DNA during stress, physically shielding DNA from reactive agents. In the late stationary phase, Dps forms higher-order structures with the nucleoids such as toroids, coral reef,^69^ and cocrystals to tailor transcriptional responses at the same time as protecting DNA from damage.^70^

**Figure 6:**
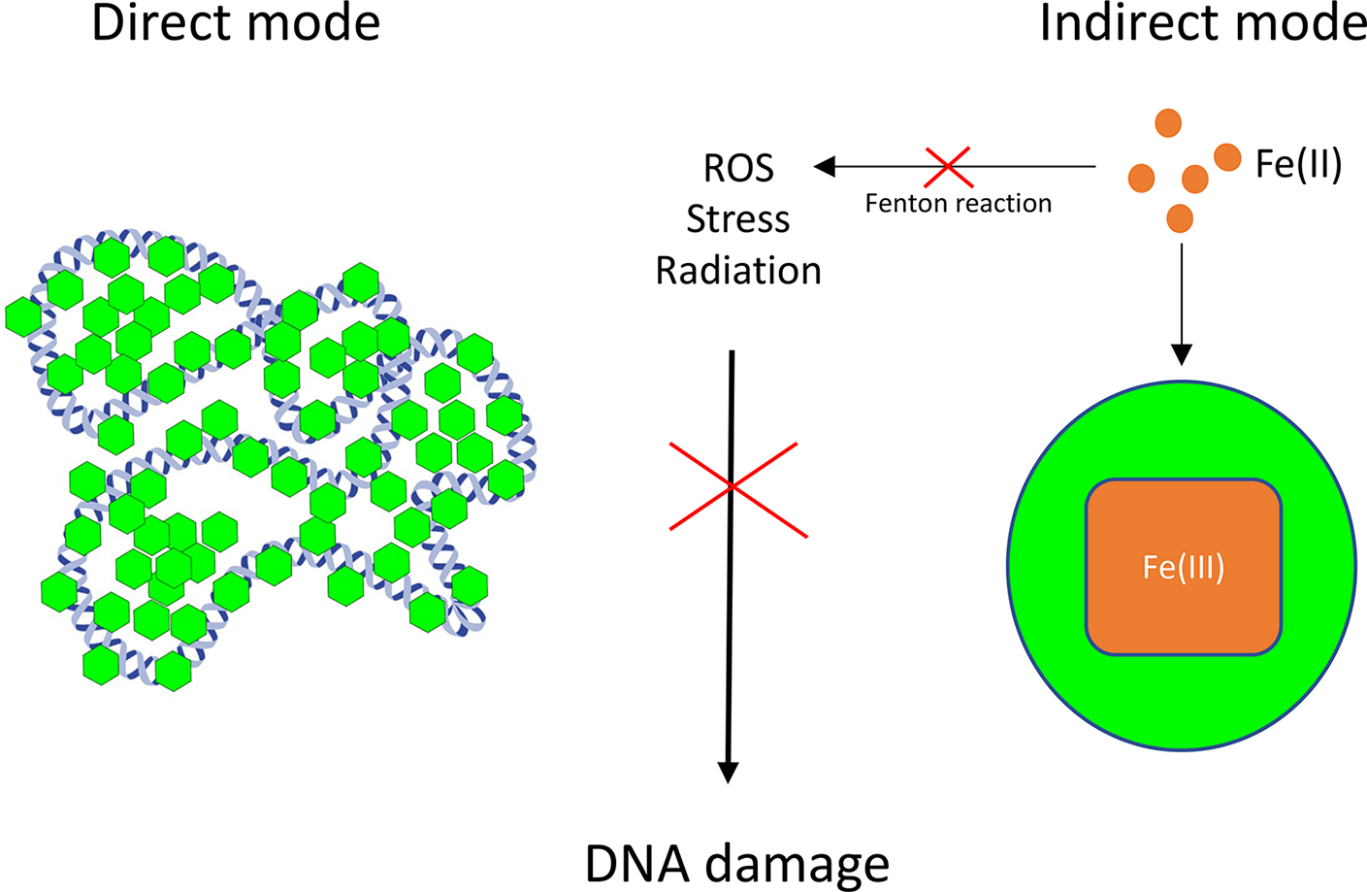
DNA protection by Dps involves a direct mode by physical binding and condensation of DNA, protecting it from the onslaught of deleterious agents. An indirect mode of action is through the sequestration of free iron, preventing the generation of reactive oxygen species (ROS) by the Fenton reaction.

Another indirect mode of protection is by sequestering iron and preventing ferrous iron from participating in the Fenton reaction (Reaction 2), which checks the formation of free radicals deleterious to DNA. The ferroxidation activity of Dps also removes toxic peroxides by using H_2_O_2_ as an oxidant. Thus, Dps utilize dual modes to afford protection to cells from multiple stresses and preserve the integrity of DNA (Figure 6).

Dps lack any known DNA binding motifs, but there is some consensus about how DNA binding is achieved by Dps. In *E. coli* Dps, the DNA binding is attributed to the lysine-rich N-terminus.^71^ In certain Dps homologs like the second Dps from *M. smegmatis* MsDps2, lacking any N- or C-terminal tails, DNA binding is achieved by residues at the N-termini from adjacent dodecamers which line the intermolecular spaces between the hexagonally closed packed layers of MsDps2 molecules.^72^ This is consistent with the recent subtomogram reconstructions of Dps-DNA cocrystals in two different lattices, where each Dps is surrounded by four or six DNA strands threading through the space between the Dps molecules.^73^ However, the exact nature of binding and the residues stabilizing the Dps-DNA interactions could not be revealed in the low-resolution tomography reconstructions. Recently, with the use of single-particle cryoEM, Garg et al. identified several arginine residues in MsDps2 lining the interstitial space of the MsDps2 hexagonal lattice which was shown to interact with DNA.^74^

It is to be noted that not all Dps have been shown to bind DNA. *A. tumefaciens* Dps has a truncated N-terminus which is fixed on the protein surface and not available for DNA binding.^75^*L. innocua* Dps does not bind DNA but has been shown to protect DNA from cleavage assays through its ferroxidation property.^76^ Streptococcal Dpr proteins from *S. suis*, *S. pyogenes*, and *S. mutans* do not bind DNA but provide protection from oxidative stress through sequestration and oxidation of iron.^48^*Bacillus anthracis* has two Dps homologues, BaDps1 and BaDps2, acting as a pair to render protection from peroxide stress without having DNA binding ability.^77^ A neutrophil activating protein from *H. pylori* is a Dps homologue termed HP-NAP that binds DNA only in its iron-loaded form.^78^

### Regulation of Dps in Cells

The vast majority of Dps proteins is expressed in bacterial cells in response to oxidative or nutritional stress. In *E. coli*, Dps expression is dependent on the phase of growth and oxidative stress. OxyR induces Dps expression in the exponential phase in response to H_2_O_2_ exposure, whereas in the stationary phase, σ^S^ (the stationary phase sigma factor) induces Dps expression.

*E. coli* Dps expression is downregulated at the promoter level during the exponential phase and in the absence of oxidative stress by nucleoid associated proteins Fis and H-NS.^79,80^ During this stage, Dps is present at its lowest number of around 6000 Dps molecules per cell (compared to the late stationary phase when it reaches a peak of 180,000 molecules per cell^81^), when Fis and H-NS levels are highest. Fis and H-NS bind at adjacent sites of the Dps core promoter and repress its expression by preventing transcription initiation by the σ^70^ RNA polymerase. The repression by H-NS can be overcome by the σ^S^ RNA polymerase. On the other hand, Fis binds at the spacer region between −10 and −35, trapping the σ^70^ RNA polymerase and forming a closed complex with the polymerase.^50^ But as in the case of H-NS, the downregulation by Fis cannot be overcome by σ^S^ RNA polymerase.^82^

Dps are also regulated post transcriptionally in cells via proteolysis. Typically, *E. coli* Dps levels are high in the stationary phase, and they are rapidly degraded during the exponential phase by the proteases ClpX/ClpP^83^ and ClpS/ClpA/ClpP.^84^ The N-terminus of *E. coli* Dps harbors ClpX and ClpS recognition motifs which promotes degradation by ClpP and ClpA/ClpP proteases, respectively.

Some organisms with more than one Dps homologue exhibit differential regulation as was seen in the case of two Dps homologues MsDps1 and MsDps2 from *M. smegmatis.* MsDps1 is overexpressed during nutritional starvation or the stationary phase with protein expression being driven by the ECF sigma factors (σ^F^ and σ^H^). MsDps2 levels seem to be constant in cells and constitutively expressed by housekeeping sigma factors (σ^A^ and σ^B^).^85,45^

## 4 Interplay between FE–S Cluster Proteins and Ferritin-Like Proteins

Iron–sulfur (Fe–S) proteins are responsible for vital processes for sustaining life, namely, photosynthesis, respiration, and nitrogen fixation. Fe–S proteins are involved in a variety of cellular functions, and therefore defective assembly of Fe–S proteins could result in global metabolic defects or cell death. These proteins get their name due to the presence of iron–sulfur clusters containing sulfide linked to di-, tri-, and tetrairon centers in variable oxidation states. Fe–S clusters are possibly the most abundant and diversly employed of the enzymatic cofactors.^86^ More commonly, Fe–S clusters have two, three, or four iron atoms coordinated to polypeptide residues bridged by inorganic sulfides.^87^ In accordance with the strong affinity of iron for thiolates, cysteine is by far the most common amino acid ligand, but histidine, aspartate, and arginine have also been observed.^88^

Before the advent of oxygen, the biosphere was driven by anaerobic metabolisms where iron and sulfur were plentiful and recruited within ancient proteins such as Fe–S cluster proteins. However, with the arrival of oxygen in the Earth’s atmosphere by photosynthetic organisms, there was an immediate threat to cluster-dependent proteins. In the presence of oxygen, as described earlier, iron is oxidized to its nonbioavailable ferric form. Bacteria have extraordinary requirements of intracellular iron^89^ as they are an essential cofactor in many enzymatic reactions. Therefore, in aerobic environments, the bioavailability of iron poses a huge problem.

Also, ROS as a byproduct of oxygen metabolism leads to the decomposition of Fe–S clusters by converting them into unstable forms. Aerobes due to their overwhelming reliance on Fe–S clusters were therefore vulnerable to iron restriction and oxidative stress. Understanding the mechanisms behind the regulation of iron delivery to Fe–S clusters and repair of damaged Fe–S clusters is crucial to understanding Fe–S proteins in the context of aerobic microbes.

### Iron Storage Proteins as Donors in Fe–S Cluster Biosynthesis

Three mechanisms for Fe–S cluster biogenesis have been identified, namely, ISC (iron–sulfur cluster), nitrogen fixing (NIF), and S utilization factor (SUF) mechanisms.^90,91^ The NIF pathway is specific to the assembly of Fe–S clusters for nitrogenase and was the first discovered system.^92^ The *isc* gene region was identified in *A. vinelandii*, and its products have a house keeping role in Fe–S cluster assembly and are distributed across almost all domains of life.^91,93^ The Suf system is usually expressed in response to conditions of oxidative stress or Fe starvation^94,95^ and is the major system of Fe–S cluster biosynthesis in Cyanobacteria.^2,95,96^ Recently two additional “minimal” Fe–S cluster assembly machineries, namely, MIS (minimal iron–sulfur) and SMS (SUF-like minimal system), were identified by Garcia et al., through homology searches with genomic context analysis and phylogeny.^97^ Mapping of the five Fe–S biogenesis systems onto phylogenies of bacteria and archaea showed that SUF and SMS are more widespread, whereas ISC and NIF are limited to a few bacterial clades.

Iron storage proteins, primarily those of the ferritin family, have been proposed to play a role in regulating iron reserves at optimal levels in cells. The ferritin-like iron storage proteins of cyanobacteria, namely, ferritins, bacterioferritins, and Dps, are thought to play an important role in iron homeostasis and could have a role in regulating iron availability in Fe–S cluster biosynthesis.^98^ In *E. coli*, the Suf pathway assembles Fe–S clusters during conditions of iron starvation and oxidative stress. Dps, which is overexpressed during starvation conditions in *E. coli*, has been shown to play a role in the *in vivo* donation of iron to the Suf pathway. In *E. coli* strains with double deletions of both Bfr and Dps, the Suf system was not efficient in performing Fe–S cluster biogenesis under nutritional stress. Deletion of FtnB and Bfr caused a similar inability for the mutant strains to synthesize Fe–S clusters under iron limitation due to an impaired Suf pathway. Therefore, it is proposed that Bfr, Dps, and FtnB are sources of iron for the Suf Fe–S cluster biogenesis pathway and have some redundancy in their functions.^99^

Fe–S cluster proteins such as ferredoxins have been implicated in the iron release machinery of ferritin-like proteins. In *Nostoc punctiforme* ferredoxins have been shown to interact with *N. punctiforme* Dps (NpDps4 and NpDps5) *in vitro* using fluorescence correlation spectroscopy (FCS) and fluorescence resonance energy transfer (FRET). This indicates that ferredoxins are involved in cellular iron homeostasis by interacting with Dps and may mediate electron transfer for reduction to release iron from the mineral core in Dps.^32^ Bacterioferritin from *P. aeruginosa*, Pa-Bfr, requires a ferredoxin named Pa-Bfd (bacterioferritin-associated ferredoxin) for mobilization of stored iron.^31^ A crystal structure of the Pa-BfrB–Pa-Bfd complex revealed an interface that ideally positions the Pa-Bfd [2Fe-2S] cluster to transfer electrons to the heme in Pa-BfrB and support Fe^2+^ mobilization.^100^

### Fe–S Cluster: Vulnerability to Oxidants

Fe–S proteins are a prime target for oxidative stress due to their Fe–S cluster redox center. In the 1990s it was discovered that superoxide inactivates the [4Fe-4S] family of dehydratases, including key enzymes of the branched-chain and TCA pathways.^101^ The damage was attributed to the superoxide directly oxidizing the Fe–S cluster, converting the [4Fe-4S]^2+^ form to an unstable [4Fe-4S]^3+^ state, which releases iron.^5^ The resultant [3Fe-4S]^1+^ cluster lacks a catalytic iron atom and renders the enzyme inactive. H_2_O_2_ has been shown to oxidize these clusters in a similar fashion.^102^

During peroxide exposure, OxyR induces the expression of several genes to combat oxidative stress. One of the ways OxyR acts to minimize the levels of free iron in the cell is by inducing the production of Dps which sequesters unincorporated iron. Dps also scavenges iron from damaged iron clusters, minimizing the formation of hydroxyl radicals. OxyR also induces the expression of a Fur repressor, which leads to a lowered synthesis of iron importers.^103^ Iron storage proteins like ferritins, Bfrs, and Dps are a potential source of Fe required for repair of oxidatively damaged Fe–S clusters. In *E. coli* ferritin A and Bfr function sequentially to provide iron required to repair [4Fe-4S] dehydratase clusters of 6-phosphogluconate dehydratase.^104^ In *Salmonella enterica* sv. Typhimurium ferritin B was specifically implicated in iron–sulfur cluster repair.^105^

YtfE, the RIC (repair of iron clusters), is a di-iron hemerythrin-like protein that functions to repair stress-damaged Fe–S clusters.^106^ The Fe atoms of holo-YtfE are labile and can be utilized as a source of iron for Fe–S cluster repair. YtfE interacts with the Fe scavenger Dps *in vivo*, as was shown by two-hybrid screening to search for interaction partners for the RIC protein. This led to the possibility of Dps as a provider of Fe to YtfE and the reconstitution of the di-iron cluster in YtfE to be used for repair of damaged Fe–S clusters.^107^ In *S. aureus*, YtfE and Dps are known to protect against H_2_O_2_ damage and are regulated by the same SrrAB pathway.

## 5 Concluding Remarks

**Figure 7:**
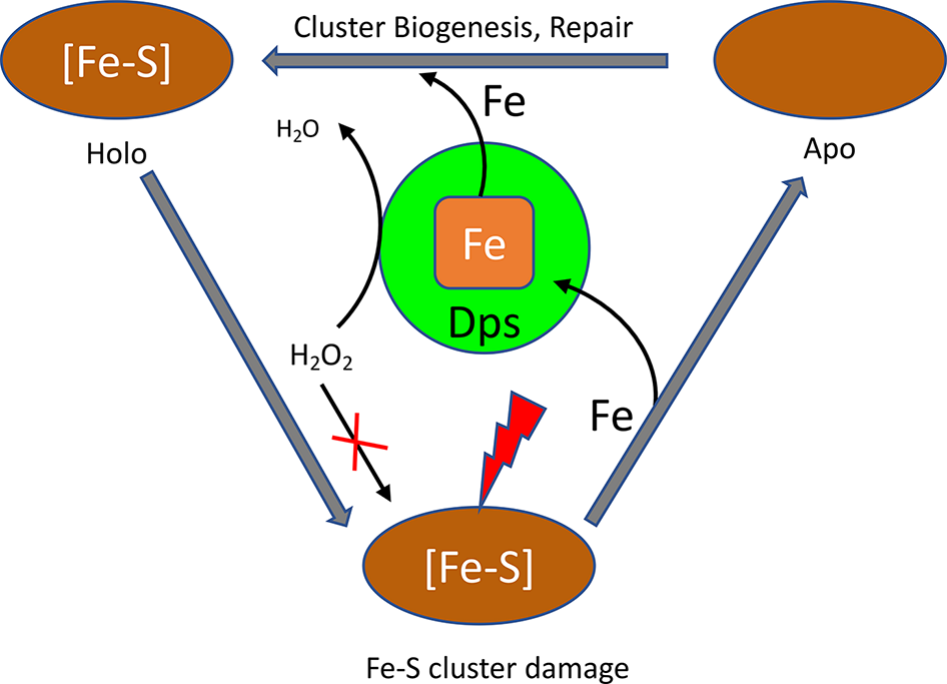
Role of Dps in Fe–S cluster protein maintenance. The Fe–S cluster protein is denoted by a brown oval with holo [Fe–S] and apo forms. Dps is shown as a green sphere and could be involved in donating iron to the biosynthesis\repair pathways. It also prevents the Fenton-mediated generation of ROS and prevents oxidative damage to the cluster.

Fe–S cluster proteins are essential in many biological processes. Despite much progress in Fe–S cluster biosynthesis/repair pathways, there is a dearth of structural information on many proteins involved in this process. Some of the key components of iron and electron donors and the molecular mechanisms behind this are relatively unknown. Since Dps are key components of iron homeostasis in bacteria, they are highly possible candidates for the maintenance of these clusters. The potential routes by which Dps could aspect Fe–S proteins are summarized in Figure 7.

## References

[ref1] Andrews S. C.; Robinson A. K.; Rodriguez-Quinones F. Bacterial iron homeostasis. FEMS Microbiol Rev. 2003, 27 (2–3), 215–237. 10.1016/S0168-6445(03)00055-X. 12829269

[ref2] Ayala-Castro C.; Saini A.; Outten F. W. Fe-S cluster assembly pathways in bacteria. Microbiol Mol. Biol. Rev. 2008, 72 (1), 110–125. 10.1128/MMBR.00034-07. 18322036 PMC2268281

[ref3] Storz G.; Imlayt J. A Oxidative stress. Curr. Opin Microbiol 1999, 2 (2), 188–194. 10.1016/S1369-5274(99)80033-2. 10322176

[ref4] Frawley E. R.; Fang F. C. The ins and outs of bacterial iron metabolism. Mol. Microbiol. 2014, 93 (4), 609–616. 10.1111/mmi.12709. 25040830 PMC4135372

[ref5] Imlay J. A. Iron-sulphur clusters and the problem with oxygen. Mol. Microbiol. 2006, 59 (4), 1073–1082. 10.1111/j.1365-2958.2006.05028.x. 16430685

[ref6] Galaris D.; Barbouti A.; Pantopoulos K. Iron homeostasis and oxidative stress: An intimate relationship. Biochim Biophys Acta Mol. Cell Res. 2019, 1866 (12), 118535 10.1016/j.bbamcr.2019.118535. 31446062

[ref7] Bradley J. M.; Svistunenko D. A.; Wilson M. T.; Hemmings A. M.; Moore G. R.; Le Brun N. E. Bacterial iron detoxification at the molecular level. J. Biol. Chem. 2020, 295 (51), 17602–17623. 10.1074/jbc.REV120.007746. 33454001 PMC7762939

[ref8] Imlay J. A. Pathways of oxidative damage. Annu. Rev. Microbiol. 2003, 57, 395–418. 10.1146/annurev.micro.57.030502.090938. 14527285

[ref9] Martinez A.; Kolter R. Protection of DNA during oxidative stress by the nonspecific DNA-binding protein Dps. J. Bacteriol. 1997, 179 (16), 5188–5194. 10.1128/jb.179.16.5188-5194.1997. 9260963 PMC179379

[ref10] de Mello Filho A. C.; Meneghini R. Protection of mammalian cells by o-phenanthroline from lethal and DNA-damaging effects produced by active oxygen species. Biochim. Biophys. Acta 1985, 847 (1), 82–89. 10.1016/0167-4889(85)90156-9. 2996616

[ref11] Cabantchik Z. I. Labile iron in cells and body fluids: physiology, pathology, and pharmacology. Front Pharmacol 2014, 5, 45 10.3389/fphar.2014.00045. 24659969 PMC3952030

[ref12] Gunther M. R.; Hanna P. M.; Mason R. P.; Cohen M. S. Hydroxyl radical formation from cuprous ion and hydrogen peroxide: a spin-trapping study. Arch. Biochem. Biophys. 1995, 316 (1), 515–522. 10.1006/abbi.1995.1068. 7840659

[ref13] Chung C. Y.; Posimo J. M.; Lee S.; Tsang T.; Davis J. M.; Brady D. C.; Chang C. J. Activity-based ratiometric FRET probe reveals oncogene-driven changes in labile copper pools induced by altered glutathione metabolism. Proc. Natl. Acad. Sci. U. S. A. 2019, 116 (37), 18285–18294. 10.1073/pnas.1904610116. 31451653 PMC6744846

[ref14] Ilari A.; Ceci P.; Ferrari D.; Rossi G. L.; Chiancone E. Iron incorporation into Escherichia coli Dps gives rise to a ferritin-like microcrystalline core. J. Biol. Chem. 2002, 277 (40), 37619–37623. 10.1074/jbc.M206186200. 12163499

[ref15] Compan I.; Touati D. Interaction of six global transcription regulators in expression of manganese superoxide dismutase in Escherichia coli K-12. J. Bacteriol. 1993, 175 (6), 1687–1696. 10.1128/jb.175.6.1687-1696.1993. 8449876 PMC203963

[ref16] Abdul-Tehrani H.; Hudson A. J.; Chang Y. S.; Timms A. R.; Hawkins C.; Williams J. M.; Harrison P. M.; Guest J. R.; Andrews S. C. Ferritin mutants of Escherichia coli are iron deficient and growth impaired, and fur mutants are iron deficient. J. Bacteriol. 1999, 181 (5), 1415–1428. 10.1128/JB.181.5.1415-1428.1999. 10049371 PMC93529

[ref17] Guerinot M. L. Microbial iron transport. Annu. Rev. Microbiol. 1994, 48, 743–772. 10.1146/annurev.mi.48.100194.003523. 7826025

[ref18] Hider R. C.; Kong X. Chemistry and biology of siderophores. Nat. Prod Rep 2010, 27 (5), 637–657. 10.1039/b906679a. 20376388

[ref19] Braun V.; Hantke K. Recent insights into iron import by bacteria. Curr. Opin Chem. Biol. 2011, 15 (2), 328–334. 10.1016/j.cbpa.2011.01.005. 21277822

[ref20] Schalk I. J.; Hannauer M.; Braud A. New roles for bacterial siderophores in metal transport and tolerance. Environ. Microbiol 2011, 13 (11), 2844–2854. 10.1111/j.1462-2920.2011.02556.x. 21883800

[ref21] Miethke M. Molecular strategies of microbial iron assimilation: from high-affinity complexes to cofactor assembly systems. Metallomics 2013, 5 (1), 15–28. 10.1039/C2MT20193C. 23192658

[ref22] Grosse C.; Scherer J.; Koch D.; Otto M.; Taudte N.; Grass G. A new ferrous iron-uptake transporter, EfeU (YcdN), from Escherichia coli. Mol. Microbiol. 2006, 62 (1), 120–131. 10.1111/j.1365-2958.2006.05326.x. 16987175

[ref23] Hantke K. Is the bacterial ferrous iron transporter FeoB a living fossil?. Trends Microbiol 2003, 11 (5), 192–195. 10.1016/S0966-842X(03)00100-8. 12781516

[ref24] Lau C. K.; Krewulak K. D.; Vogel H. J. Bacterial ferrous iron transport: the Feo system. FEMS Microbiol Rev. 2016, 40 (2), 273–298. 10.1093/femsre/fuv049. 26684538

[ref25] Sheldon J. R.; Laakso H. A.; Heinrichs D. E. Iron Acquisition Strategies of Bacterial Pathogens. Microbiol Spectr 2016, 10.1128/microbiolspec.VMBF-0010-2015. 27227297

[ref26] Theil E. C.; Tosha T.; Behera R. K. Solving Biology’s Iron Chemistry Problem with Ferritin Protein Nanocages. Acc. Chem. Res. 2016, 49 (5), 784–791. 10.1021/ar500469e. 27136423

[ref27] Williams S. M.; Chatterji D. An Overview of Dps: Dual Acting Nanovehicles in Prokaryotes with DNA Binding and Ferroxidation Properties. Subcell Biochem 2021, 96, 177–216. 10.1007/978-3-030-58971-4_3. 33252729

[ref28] Ilari A.; Stefanini S.; Chiancone E.; Tsernoglou D. The dodecameric ferritin from Listeria innocua contains a novel intersubunit iron-binding site. Nat. Struct. Biol. 2000, 7 (1), 38–43. 10.1038/71236. 10625425

[ref29] Theil E. C. Ferritin: the protein nanocage and iron biomineral in health and in disease. Inorg. Chem. 2013, 52 (21), 12223–12233. 10.1021/ic400484n. 24102308 PMC3882016

[ref30] Topham R.; Goger M.; Pearce K.; Schultz P. The mobilization of ferritin iron by liver cytosol. A comparison of xanthine and NADH as reducing substrates. Biochem. J. 1989, 261 (1), 137–143. 10.1042/bj2610137. 2775199 PMC1138793

[ref31] Eshelman K.; Yao H.; Punchi Hewage A. N. D.; Deay J. J.; Chandler J. R.; Rivera M. Inhibiting the BfrB:Bfd interaction in Pseudomonas aeruginosa causes irreversible iron accumulation in bacterioferritin and iron deficiency in the bacterial cytosol. Metallomics 2017, 9 (6), 646–659. 10.1039/C7MT00042A. 28318006 PMC5494978

[ref32] Moparthi V. K.; Moparthi S. B.; Howe C.; Raleiras P.; Wenger J.; Stensjo K. Structural diffusion properties of two atypical Dps from the cyanobacterium Nostoc punctiforme disclose interactions with ferredoxins and DNA. Biochim Biophys Acta Bioenerg 2019, 1860 (10), 148063 10.1016/j.bbabio.2019.148063. 31419396

[ref33] He D.; Hughes S.; Vanden-Hehir S.; Georgiev A.; Altenbach K.; Tarrant E.; Mackay C. L.; Waldron K. J.; Clarke D. J.; Marles-Wright J. Structural characterization of encapsulated ferritin provides insight into iron storage in bacterial nanocompartments. Elife 2016, 10.7554/eLife.18972. PMC5012862 27529188

[ref34] Giessen T. W.; Silver P. A. Widespread distribution of encapsulin nanocompartments reveals functional diversity. Nat. Microbiol 2017, 2, 17029 10.1038/nmicrobiol.2017.29. 28263314

[ref35] Gort A. S.; Ferber D. M.; Imlay J. A. The regulation and role of the periplasmic copper, zinc superoxide dismutase of Escherichia coli. Mol. Microbiol. 1999, 32 (1), 179–191. 10.1046/j.1365-2958.1999.01343.x. 10216871

[ref36] Cabiscol E.; Tamarit J.; Ros J. Oxidative stress in bacteria and protein damage by reactive oxygen species. Int. Microbiol 2000, 3 (1), 3–8. 10963327

[ref37] Gu M.; Imlay J. A. The SoxRS response of Escherichia coli is directly activated by redox-cycling drugs rather than by superoxide. Mol. Microbiol. 2011, 79 (5), 1136–1150. 10.1111/j.1365-2958.2010.07520.x. 21226770 PMC3071027

[ref38] Hidalgo E.; Ding H.; Demple B. Redox signal transduction via iron-sulfur clusters in the SoxR transcription activator. Trends Biochem. Sci. 1997, 22 (6), 207–210. 10.1016/S0968-0004(97)01068-2. 9204707

[ref39] Glanville D. G.; Han L.; Maule A. F.; Woodacre A.; Thanki D.; Abdullah I. T.; Morrissey J. A.; Clarke T. B.; Yesilkaya H.; Silvaggi N. R.; et al. RitR is an archetype for a novel family of redox sensors in the streptococci that has evolved from two-component response regulators and is required for pneumococcal colonization. PLoS Pathog 2018, 14 (5), e1007052 10.1371/journal.ppat.1007052. 29750817 PMC5965902

[ref40] Frawley E. R.; Crouch M. L.; Bingham-Ramos L. K.; Robbins H. F.; Wang W.; Wright G. D.; Fang F. C. Iron and citrate export by a major facilitator superfamily pump regulates metabolism and stress resistance in Salmonella Typhimurium. Proc. Natl. Acad. Sci. U. S. A. 2013, 110 (29), 12054–12059. 10.1073/pnas.1218274110. 23821749 PMC3718157

[ref41] Sankari S.; O’Brian M. R. A bacterial iron exporter for maintenance of iron homeostasis. J. Biol. Chem. 2014, 289 (23), 16498–16507. 10.1074/jbc.M114.571562. 24782310 PMC4047416

[ref42] Ruangkiattikul N.; Bhubhanil S.; Chamsing J.; Niamyim P.; Sukchawalit R.; Mongkolsuk S. Agrobacterium tumefaciens membrane-bound ferritin plays a role in protection against hydrogen peroxide toxicity and is negatively regulated by the iron response regulator. FEMS Microbiol Lett. 2012, 329 (1), 87–92. 10.1111/j.1574-6968.2012.02509.x. 22268462

[ref43] Bhubhanil S.; Chamsing J.; Sittipo P.; Chaoprasid P.; Sukchawalit R.; Mongkolsuk S. Roles of Agrobacterium tumefaciens membrane-bound ferritin (MbfA) in iron transport and resistance to iron under acidic conditions. Microbiology (Reading) 2014, 160 (5), 863–871. 10.1099/mic.0.076802-0. 24600024

[ref44] Gupta S.; Chatterji D. Bimodal protection of DNA by Mycobacterium smegmatis DNA-binding protein from stationary phase cells. J. Biol. Chem. 2003, 278 (7), 5235–5241. 10.1074/jbc.M208825200. 12466274

[ref45] Chowdhury R. P.; Saraswathi R.; Chatterji D. Mycobacterial stress regulation: The Dps ″twin sister″ defense mechanism and structure-function relationship. IUBMB Life 2009, 62 (1), 67–77. 10.1002/iub.285. 20014234

[ref46] Nair S.; Finkel S. E. Dps protects cells against multiple stresses during stationary phase. J. Bacteriol. 2004, 186 (13), 4192–4198. 10.1128/JB.186.13.4192-4198.2004. 15205421 PMC421617

[ref47] Yamamoto Y.; Poole L. B.; Hantgan R. R.; Kamio Y. An iron-binding protein, Dpr, from Streptococcus mutans prevents iron-dependent hydroxyl radical formation in vitro. J. Bacteriol. 2002, 184 (11), 2931–2939. 10.1128/JB.184.11.2931-2939.2002. 12003933 PMC135054

[ref48] Tsou C. C.; Chiang-Ni C.; Lin Y. S.; Chuang W. J.; Lin M. T.; Liu C. C.; Wu J. J. An iron-binding protein, Dpr, decreases hydrogen peroxide stress and protects Streptococcus pyogenes against multiple stresses. Infect. Immun. 2008, 76 (9), 4038–4045. 10.1128/IAI.00477-08. 18541662 PMC2519395

[ref49] Howe C.; Ho F.; Nenninger A.; Raleiras P.; Stensjo K. Differential biochemical properties of three canonical Dps proteins from the cyanobacterium Nostoc punctiforme suggest distinct cellular functions. J. Biol. Chem. 2018, 293 (43), 16635–16646. 10.1074/jbc.RA118.002425. 30171072 PMC6204913

[ref50] Calhoun L. N.; Kwon Y. M. Structure, function and regulation of the DNA-binding protein Dps and its role in acid and oxidative stress resistance in Escherichia coli: a review. J. Appl. Microbiol. 2011, 110 (2), 375–386. 10.1111/j.1365-2672.2010.04890.x. 21143355

[ref51] Shu J. C.; Soo P. C.; Chen J. C.; Hsu S. H.; Chen L. C.; Chen C. Y.; Liang S. H.; Buu L. M.; Chen C. C. Differential regulation and activity against oxidative stress of Dps proteins in Bacillus cereus. Int. J. Med. Microbiol 2013, 303 (8), 662–673. 10.1016/j.ijmm.2013.09.011. 24383075

[ref52] Chen Y.; Yang Z.; Zhou X.; Jin M.; Dai Z.; Ming D.; Zhang Z.; Zhu L.; Jiang L. Sequence, structure, and function of the Dps DNA-binding protein from Deinococcus wulumuqiensis R12. Microb Cell Fact 2022, 21 (1), 132 10.1186/s12934-022-01857-7. 35780107 PMC9250271

[ref53] Ueshima J.; Shoji M.; Ratnayake D. B.; Abe K.; Yoshida S.; Yamamoto K.; Nakayama K. Purification, gene cloning, gene expression, and mutants of Dps from the obligate anaerobe Porphyromonas gingivalis. Infect. Immun. 2003, 71 (3), 1170–1178. 10.1128/IAI.71.3.1170-1178.2003. 12595429 PMC148816

[ref54] Karas V. O.; Westerlaken I.; Meyer A. S. The DNA-Binding Protein from Starved Cells (Dps) Utilizes Dual Functions To Defend Cells against Multiple Stresses. J. Bacteriol. 2015, 197 (19), 3206–3215. 10.1128/JB.00475-15. 26216848 PMC4560292

[ref55] Zhao G.; Ceci P.; Ilari A.; Giangiacomo L.; Laue T. M.; Chiancone E.; Chasteen N. D. Iron and hydrogen peroxide detoxification properties of DNA-binding protein from starved cells. A ferritin-like DNA-binding protein of Escherichia coli. J. Biol. Chem. 2002, 277 (31), 27689–27696. 10.1074/jbc.M202094200. 12016214

[ref56] Bou-Abdallah F.; Yang H.; Awomolo A.; Cooper B.; Woodhall M. R.; Andrews S. C.; Chasteen N. D. Functionality of the three-site ferroxidase center of Escherichia coli bacterial ferritin (EcFtnA). Biochemistry 2014, 53 (3), 483–495. 10.1021/bi401517f. 24380371 PMC3951517

[ref57] Bunker J.; Lowry T.; Davis G.; Zhang B.; Brosnahan D.; Lindsay S.; Costen R.; Choi S.; Arosio P.; Watt G. D. Kinetic studies of iron deposition catalyzed by recombinant human liver heavy and light ferritins and Azotobacter vinelandii bacterioferritin using O2 and H2O2 as oxidants. Biophys Chem. 2005, 114 (2–3), 235–244. 10.1016/j.bpc.2004.11.008. 15829358

[ref58] Pullin J.; Wilson M. T.; Clemancey M.; Blondin G.; Bradley J. M.; Moore G. R.; Le Brun N. E.; Lucic M.; Worrall J. A. R.; Svistunenko D. A. Iron Oxidation in Escherichia coli Bacterioferritin Ferroxidase Centre, a Site Designed to React. Rapidly with H(2) O(2) but Slowly with O(2). Angew. Chem., Int. Ed. Engl. 2021, 60 (15), 8361–8369. 10.1002/anie.202015964. 33482043 PMC8049013

[ref59] Williams S. M.; Chandran A. V.; Prakash S.; Vijayan M.; Chatterji D. A Mutation Directs the Structural Switch of DNA Binding Proteins under Starvation to a Ferritin-like Protein Cage. Structure 2017, 25 (9), 1449–1454. 10.1016/j.str.2017.07.006. 28823472

[ref60] Andrews S. C. The Ferritin-like superfamily: Evolution of the biological iron storeman from a rubrerythrin-like ancestor. Biochim. Biophys. Acta 2010, 1800 (8), 691–705. 10.1016/j.bbagen.2010.05.010. 20553812

[ref61] Pulliainen A. T.; Kauko A.; Haataja S.; Papageorgiou A. C.; Finne J. Dps/Dpr ferritin-like protein: insights into the mechanism of iron incorporation and evidence for a central role in cellular iron homeostasis in Streptococcus suis. Mol. Microbiol. 2005, 57 (4), 1086–1100. 10.1111/j.1365-2958.2005.04756.x. 16091046

[ref62] Bellapadrona G.; Stefanini S.; Zamparelli C.; Theil E. C.; Chiancone E. Iron translocation into and out of Listeria innocua Dps and size distribution of the protein-enclosed nanomineral are modulated by the electrostatic gradient at the 3-fold ″ferritin-like″ pores. J. Biol. Chem. 2009, 284 (28), 19101–19109. 10.1074/jbc.M109.014670. 19457858 PMC2707210

[ref63] Williams S. M.; Chandran A. V.; Vijayabaskar M. S.; Roy S.; Balaram H.; Vishveshwara S.; Vijayan M.; Chatterji D. A histidine aspartate ionic lock gates the iron passage in miniferritins from Mycobacterium smegmatis. J. Biol. Chem. 2014, 289 (16), 11042–11058. 10.1074/jbc.M113.524421. 24573673 PMC4036245

[ref64] Williams S. M.; Chatterji D. Flexible aspartates propel iron to the ferroxidation sites along pathways stabilized by a conserved arginine in Dps proteins from Mycobacterium smegmatis. Metallomics 2017, 9 (6), 685–698. 10.1039/C7MT00008A. 28418062

[ref65] Arnold A. R.; Zhou A.; Barton J. K. Characterization of the DNA-Mediated Oxidation of Dps, A Bacterial Ferritin. J. Am. Chem. Soc. 2016, 138 (35), 11290–11298. 10.1021/jacs.6b06507. 27571139 PMC5014645

[ref66] Chesnokov Y.; Mozhaev A.; Kamyshinsky R.; Gordienko A.; Dadinova L. Structural Insights into Iron Ions Accumulation in Dps Nanocage. Int. J. Mol. Sci. 2022, 23 (10), 5313 10.3390/ijms23105313. 35628121 PMC9140674

[ref67] Chen J. H.; Hales C. N.; Ozanne S. E. DNA damage, cellular senescence and organismal ageing: causal or correlative?. Nucleic Acids Res. 2007, 35 (22), 7417–7428. 10.1093/nar/gkm681. 17913751 PMC2190714

[ref68] Wolf S. G.; Frenkiel D.; Arad T.; Finkel S. E.; Kolter R.; Minsky A. DNA protection by stress-induced biocrystallization. Nature 1999, 400 (6739), 83–85. 10.1038/21918. 10403254

[ref69] Ghatak P.; Karmakar K.; Kasetty S.; Chatterji D. Unveiling the role of Dps in the organization of mycobacterial nucleoid. PLoS One 2011, 6 (1), e16019 10.1371/journal.pone.0016019. 21283627 PMC3026007

[ref70] Janissen R.; Arens M. M. A.; Vtyurina N. N.; Rivai Z.; Sunday N. D.; Eslami-Mossallam B.; Gritsenko A. A.; Laan L.; de Ridder D.; Artsimovitch I.; et al. Global DNA Compaction in Stationary-Phase Bacteria Does Not Affect Transcription. Cell 2018, 174 (5), 1188–1199. 10.1016/j.cell.2018.06.049. 30057118 PMC6108918

[ref71] Ceci P.; Cellai S.; Falvo E.; Rivetti C.; Rossi G. L.; Chiancone E. DNA condensation and self-aggregation of Escherichia coli Dps are coupled phenomena related to the properties of the N-terminus. Nucleic Acids Res. 2004, 32 (19), 5935–5944. 10.1093/nar/gkh915. 15534364 PMC528800

[ref72] Roy S.; Saraswathi R.; Gupta S.; Sekar K.; Chatterji D.; Vijayan M. Role of N and C-terminal tails in DNA binding and assembly in Dps: structural studies of Mycobacterium smegmatis Dps deletion mutants. J. Mol. Biol. 2007, 370 (4), 752–767. 10.1016/j.jmb.2007.05.004. 17543333

[ref73] Kamyshinsky R.; Chesnokov Y.; Dadinova L.; Mozhaev A.; Orlov I.; Petoukhov M.; Orekhov A.; Shtykova E.; Vasiliev A. Polymorphic Protective Dps-DNA Co-Crystals by Cryo Electron Tomography and Small Angle X-Ray Scattering. Biomolecules 2020, 10 (1), 39 10.3390/biom10010039. PMC7023142 31888079

[ref74] Garg P.; Satheesh T.; Ganji M.; Dutta S. Cryo-EM reveals the mechanism of DNA compaction by Mycobacterium smegmatis Dps2. bioRxiv 2023, 10.1101/2023.01.16.523357. 39349276

[ref75] Ceci P.; Ilari A.; Falvo E.; Chiancone E. The Dps protein of Agrobacterium tumefaciens does not bind to DNA but protects it toward oxidative cleavage: x-ray crystal structure, iron binding, and hydroxyl-radical scavenging properties. J. Biol. Chem. 2003, 278 (22), 20319–20326. 10.1074/jbc.M302114200. 12660233

[ref76] Su M.; Cavallo S.; Stefanini S.; Chiancone E.; Chasteen N. D. The so-called Listeria innocua ferritin is a Dps protein. Iron incorporation, detoxification, and DNA protection properties. Biochemistry 2005, 44 (15), 5572–5578. 10.1021/bi0472705. 15823015

[ref77] Schwartz J. K.; Liu X. S.; Tosha T.; Diebold A.; Theil E. C.; Solomon E. I. CD and MCD spectroscopic studies of the two Dps miniferritin proteins from Bacillus anthracis: role of O2 and H2O2 substrates in reactivity of the diiron catalytic centers. Biochemistry 2010, 49 (49), 10516–10525. 10.1021/bi101346c. 21028901 PMC3075618

[ref78] Kottakis F.; Papadopoulos G.; Pappa E. V.; Cordopatis P.; Pentas S.; Choli-Papadopoulou T. Helicobacter pylori neutrophil-activating protein activates neutrophils by its C-terminal region even without dodecamer formation, which is a prerequisite for DNA protection--novel approaches against Helicobacter pylori inflammation. FEBS J. 2008, 275 (2), 302–317. 10.1111/j.1742-4658.2007.06201.x. 18076649

[ref79] Azam T. A.; Ishihama A. Twelve species of the nucleoid-associated protein from Escherichia coli. Sequence recognition specificity and DNA binding affinity. J. Biol. Chem. 1999, 274 (46), 33105–33113. 10.1074/jbc.274.46.33105. 10551881

[ref80] Grainger D. C.; Busby S. J. Global regulators of transcription in Escherichia coli: mechanisms of action and methods for study. Adv. Appl. Microbiol 2008, 65, 93–113. 10.1016/S0065-2164(08)00604-7. 19026863

[ref81] Ali Azam T.; Iwata A.; Nishimura A.; Ueda S.; Ishihama A. Growth phase-dependent variation in protein composition of the Escherichia coli nucleoid. J. Bacteriol. 1999, 181 (20), 6361–6370. 10.1128/JB.181.20.6361-6370.1999. 10515926 PMC103771

[ref82] Grainger D. C.; Goldberg M. D.; Lee D. J.; Busby S. J. Selective repression by Fis and H-NS at the Escherichia coli dps promoter. Mol. Microbiol. 2008, 68 (6), 1366–1377. 10.1111/j.1365-2958.2008.06253.x. 18452510

[ref83] Stephani K.; Weichart D.; Hengge R. Dynamic control of Dps protein levels by ClpXP and ClpAP proteases in Escherichia coli. Mol. Microbiol. 2003, 49 (6), 1605–1614. 10.1046/j.1365-2958.2003.03644.x. 12950924

[ref84] Schmidt R.; Zahn R.; Bukau B.; Mogk A. ClpS is the recognition component for Escherichia coli substrates of the N-end rule degradation pathway. Mol. Microbiol. 2009, 72 (2), 506–517. 10.1111/j.1365-2958.2009.06666.x. 19317833

[ref85] Saraswathi R.; Pait Chowdhury R.; Williams S. M.; Ghatak P.; Chatterji D. The mycobacterial MsDps2 protein is a nucleoid-forming DNA binding protein regulated by sigma factors sigma and sigma. PLoS One 2009, 4 (11), e8017 10.1371/journal.pone.0008017. 19956571 PMC2779847

[ref86] Balk J.; Schaedler T. A. Iron cofactor assembly in plants. Annu. Rev. Plant Biol. 2014, 65, 125–153. 10.1146/annurev-arplant-050213-035759. 24498975

[ref87] Rees D. C. Great metalloclusters in enzymology. Annu. Rev. Biochem. 2002, 71, 221–246. 10.1146/annurev.biochem.71.110601.135406. 12045096

[ref88] Berkovitch F.; Nicolet Y.; Wan J. T.; Jarrett J. T.; Drennan C. L. Crystal structure of biotin synthase, an S-adenosylmethionine-dependent radical enzyme. Science 2004, 303 (5654), 76–79. 10.1126/science.1088493. 14704425 PMC1456065

[ref89] Outten C. E.; O’Halloran T. V. Femtomolar sensitivity of metalloregulatory proteins controlling zinc homeostasis. Science 2001, 292 (5526), 2488–2492. 10.1126/science.1060331. 11397910

[ref90] Johnson D. C.; Dean D. R.; Smith A. D.; Johnson M. K. Structure, function, and formation of biological iron-sulfur clusters. Annu. Rev. Biochem. 2005, 74, 247–281. 10.1146/annurev.biochem.74.082803.133518. 15952888

[ref91] Lill R.; Broderick J. B.; Dean D. R. Special issue on iron-sulfur proteins: Structure, function, biogenesis and diseases. Biochim. Biophys. Acta 2015, 1853 (6), 1251–1252. 10.1016/j.bbamcr.2015.03.001. 25746719 PMC5501863

[ref92] Jacobson M. R.; Cash V. L.; Weiss M. C.; Laird N. F.; Newton W. E.; Dean D. R. Biochemical and genetic analysis of the nifUSVWZM cluster from Azotobacter vinelandii. Mol. Gen Genet 1989, 219 (1–2), 49–57. 10.1007/BF00261156. 2615765

[ref93] Zheng L.; Cash V. L.; Flint D. H.; Dean D. R. Assembly of iron-sulfur clusters. Identification of an iscSUA-hscBA-fdx gene cluster from Azotobacter vinelandii. J. Biol. Chem. 1998, 273 (21), 13264–13272. 10.1074/jbc.273.21.13264. 9582371

[ref94] Outten F. W.; Djaman O.; Storz G. A suf operon requirement for Fe-S cluster assembly during iron starvation in Escherichia coli. Mol. Microbiol. 2004, 52 (3), 861–872. 10.1111/j.1365-2958.2004.04025.x. 15101990

[ref95] Outten F. W. Recent advances in the Suf Fe-S cluster biogenesis pathway: Beyond the Proteobacteria. Biochim. Biophys. Acta 2015, 1853 (6), 1464–1469. 10.1016/j.bbamcr.2014.11.001. 25447545 PMC4390423

[ref96] Balasubramanian R.; Shen G.; Bryant D. A.; Golbeck J. H. Regulatory roles for IscA and SufA in iron homeostasis and redox stress responses in the cyanobacterium Synechococcus sp. strain PCC 7002. J. Bacteriol. 2006, 188 (9), 3182–3191. 10.1128/JB.188.9.3182-3191.2006. 16621810 PMC1447454

[ref97] Garcia P. S.; D’Angelo F.; Ollagnier de Choudens S.; Dussouchaud M.; Bouveret E.; Gribaldo S.; Barras F. An early origin of iron-sulfur cluster biosynthesis machineries before Earth oxygenation. Nat. Ecol Evol 2022, 6 (10), 1564–1572. 10.1038/s41559-022-01857-1. 36109654

[ref98] Gao F. Iron-Sulfur Cluster Biogenesis and Iron Homeostasis in Cyanobacteria. Front Microbiol 2020, 11, 165 10.3389/fmicb.2020.00165. 32184761 PMC7058544

[ref99] Bolaji N. Characterization of the SUF FE-S Pathway In Escherichia Coli. PhD Dissertation, 2017.

[ref100] Yao H.; Wang Y.; Lovell S.; Kumar R.; Ruvinsky A. M.; Battaile K. P.; Vakser I. A.; Rivera M. The structure of the BfrB-Bfd complex reveals protein-protein interactions enabling iron release from bacterioferritin. J. Am. Chem. Soc. 2012, 134 (32), 13470–13481. 10.1021/ja305180n. 22812654 PMC3428730

[ref101] Kuo C. F.; McRee D. E.; Fisher C. L.; O’Handley S. F.; Cunningham R. P.; Tainer J. A. Atomic structure of the DNA repair [4Fe-4S] enzyme endonuclease III. Science 1992, 258 (5081), 434–440. 10.1126/science.1411536. 1411536

[ref102] Varghese S.; Tang Y.; Imlay J. A. Contrasting sensitivities of Escherichia coli aconitases A and B to oxidation and iron depletion. J. Bacteriol. 2003, 185 (1), 221–230. 10.1128/JB.185.1.221-230.2003. 12486059 PMC141816

[ref103] Varghese S.; Wu A.; Park S.; Imlay K. R.; Imlay J. A. Submicromolar hydrogen peroxide disrupts the ability of Fur protein to control free-iron levels in Escherichia coli. Mol. Microbiol. 2007, 64 (3), 822–830. 10.1111/j.1365-2958.2007.05701.x. 17462026 PMC3048849

[ref104] Keyer K.; Imlay J. A. Superoxide accelerates DNA damage by elevating free-iron levels. Proc. Natl. Acad. Sci. U. S. A. 1996, 93 (24), 13635–13640. 10.1073/pnas.93.24.13635. 8942986 PMC19375

[ref105] Velayudhan J.; Castor M.; Richardson A.; Main-Hester K. L.; Fang F. C. The role of ferritins in the physiology of Salmonella enterica sv. Typhimurium: a unique role for ferritin B in iron-sulphur cluster repair and virulence. Mol. Microbiol. 2007, 63 (5), 1495–1507. 10.1111/j.1365-2958.2007.05600.x. 17302823

[ref106] Crack J. C.; Balasiny B. K.; Bennett S. P.; Rolfe M. D.; Froes A.; MacMillan F.; Green J.; Cole J. A.; Le Brun N. E. The Di-Iron Protein YtfE Is a Nitric Oxide-Generating Nitrite Reductase Involved in the Management of Nitrosative Stress. J. Am. Chem. Soc. 2022, 144 (16), 7129–7145. 10.1021/jacs.1c12407. 35416044 PMC9052748

[ref107] Silva L. S. O.; Baptista J. M.; Batley C.; Andrews S. C.; Saraiva L. M. The Di-iron RIC Protein (YtfE) of Escherichia coli Interacts with the DNA-Binding Protein from Starved Cells (Dps) To Diminish RIC Protein-Mediated Redox Stress. J. Bacteriol. 2018, 10.1128/JB.00527-18. PMC6256020 30249704

[ref108] Pettersen E. F.; Goddard T. D.; Huang C. C.; Couch G. S.; Greenblatt D. M.; Meng E. C.; Ferrin T. E. UCSF Chimera--a visualization system for exploratory research and analysis. J. Comput. Chem. 2004, 25 (13), 1605–1612. 10.1002/jcc.20084. 15264254

